# Supplementary material for: Quantitative multiplex immunohistochemistry reveals inter-patient lymphovascular and immune heterogeneity in primary cutaneous melanoma
Source: Front Immunol. 2024 Feb 1;15:1328602. doi: 10.3389/fimmu.2024.1328602 (PMC10867179; doi:10.3389/fimmu.2024.1328602)
Supplement: Supplementary Table 1 — Antibody product information. LEC: lymphatic endothelial cell. [file Table_1.pdf]

Markers	Antibody (host)	Target	Clone	Product #	Manufacturer	Dilution
Lymphovascular	Podoplanin (mouse)	PDPN; mucin-type protein, highly expressed by LECs and necessary for lymphatic development.	D240	916601	BioLegend	1:100
	LYVE-1 (rabbit)	Lymphatic vessel endothelial hyaluronan receptor 1; binds both soluble and insoluble hyaluronan, expressed by LECs.	polyclonal	ab36993	Abcam	1:100
	AQP1 (rabbit)	Aquaporin 1; widely expressed water channel showing specificity to blood over lymphatic vessels.	polyclonal	AB2219	Millipore	1:10000
	CD34 (mouse)	Sialomucin, L-selectin ligand. Critical for T lymphocyte rolling and entry into lymph nodes.	QBEnd-10	MA1-10202	Pierce (Thermo Fisher)	1:10000
	MECA79 (rat)	Binds L-selectin ligands collectively called peripheral lymph node addressins (GlyCAM-1, CD34, Agp200, and endomucin).	IgM	120801 or 2	Biolegend	1:100
	αSMA (rabbit)	Alpha smooth muscle actin; Actin isoform involved in contractile apparatus of smooth muscle.	polyclonal	ab5694	Abcam	1:500
Melanocyte	S100 (mouse)	Low molecular weight calcium binding proteins expressed by melanocytes. Also expressed in other cells of neural crest origin, macrophages, and dendritic cells.	cocktail	CM089B	Biocare Medical	1:2000
Epithelial	panCytokeratin (mouse)	Keratin proteins found in the cytoskeleton of epithelial tissue, marks epidermis.	[AE1/AE3]	Ab27988	Abcam	1:2000
Immune	CD45 (mouse)	Pan-leukocyte marker.	HI30	14-0459-82	eBioscience	1:200
	CD20 (rabbit)	B-lymphocyte antigen, expressed from late pro-B cells through memory but not plasma cells	SP32	ab64088	Abcam	1:1000
	CD8 (mouse)	Co-receptor of the T cell receptor, marker of cytotoxic T cells.	C8/144B	MS-457-S1	Thermo	1:200
	CD68 (mouse)	Transmembrane glycoprotein expressed by cells of monocyte lineage, circulating macrophages, and tissue macrophages.	PG-M1	ab783	Abcam	1:200
