# Supplementary material for: Quantitative multiplex immunohistochemistry reveals inter-patient lymphovascular and immune heterogeneity in primary cutaneous melanoma
Source: Front Immunol. 2024 Feb 1;15:1328602. doi: 10.3389/fimmu.2024.1328602 (PMC10867179; doi:10.3389/fimmu.2024.1328602)
Supplement: Supplementary Table 2 — Multiplexed staining protocol. [file Table_2.pdf]

	Retrieval	Blocking	Primary Ab	Primary Ab 2	ImmPRESS	ImmPRESS 2
**Hematoxylin**						
**Cycle 1**	Citra	Horse Serum	Mouse anti-PDPN		anti-Mouse	
**Cycle 2**	TRS	Horse Serum	Rabbit anti-LYVE-1	Rat anti-MECA79	anti-Rabbit	anti-Rat
**Cycle 3**	Citra	Horse Serum	Mouse anti-CD45		anti-Mouse	
**Cycle 4**	Citra	Horse Serum	Rabbit anti-CD20		anti-Rabbit	
**Cycle 5**	Citra	Horse Serum	Mouse anti-CD68	Rabbit anti-αSMA	anti-Mouse	anti-Rabbit
**Cycle 6**	Citra	Horse Serum	Mouse anti-S100		anti-Mouse	
**Cycle 7**	Citra	Horse Serum	Mouse anti-CD8		anti-Mouse	
**Cycle 8**	Citra	Horse Serum	Mouse anti-CD34	Rabbit anti-AQP1	anti-Mouse	anti-Rabbit
**Cycle 9**	Citra	Horse Serum	Mouse anti-panCK		anti-Mouse	
**Hematoxylin**						
