# Supplementary figures and images for: Quantitative multiplex immunohistochemistry reveals inter-patient lymphovascular and immune heterogeneity in primary cutaneous melanoma
Source: Front Immunol. 2024 Feb 1;15:1328602. doi: 10.3389/fimmu.2024.1328602 (PMC10867179; doi:10.3389/fimmu.2024.1328602)

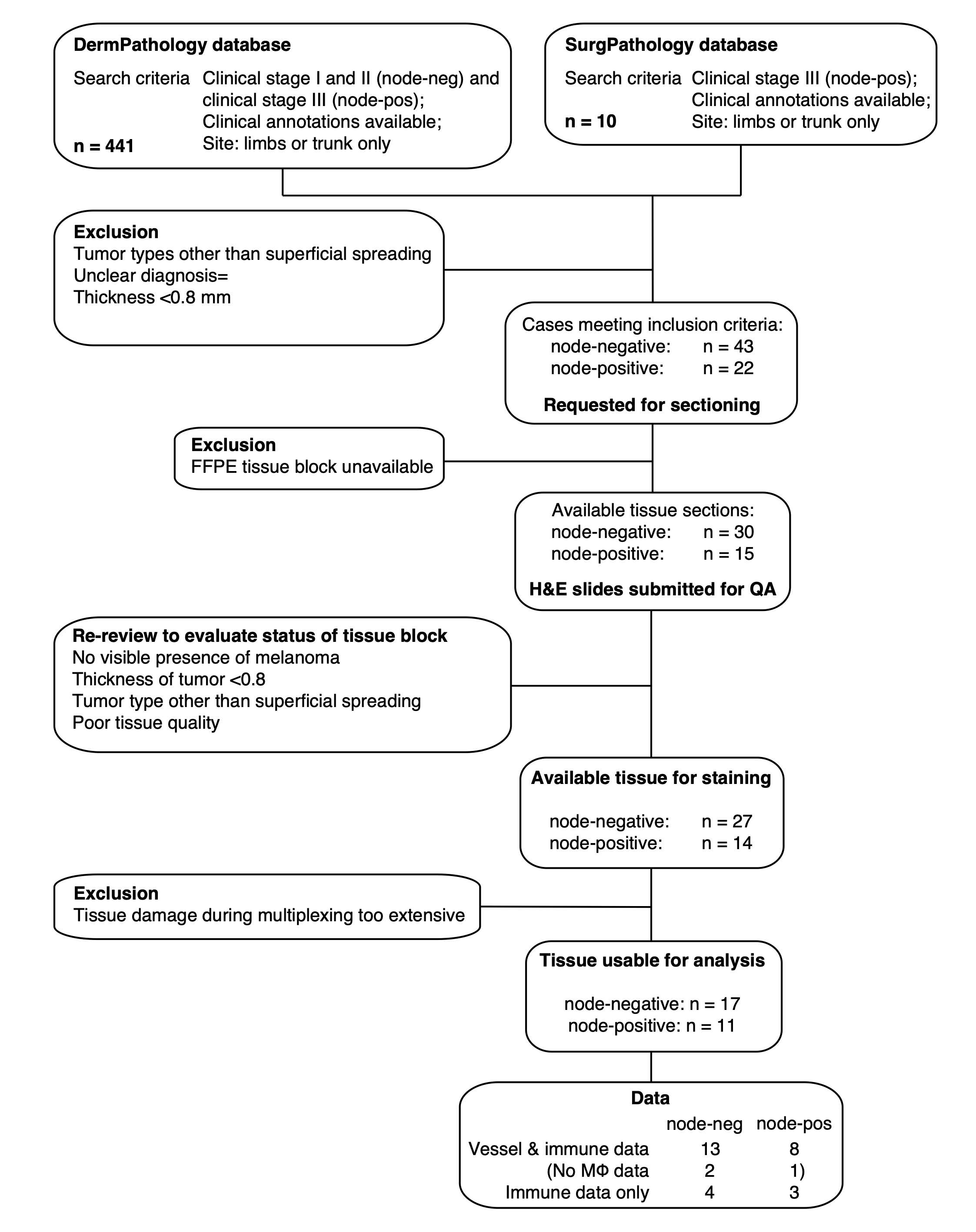

Supplement: Supplementary Figure 1 — Sample selection and validation flow chart. Sample selection decision tree including criteria for sample inclusion and exclusion. The final cohort consisted of 28 patients. Matched analysis of lymphovasculature and immune infiltrates was performed in 21 patients, with immune data only in an additional 7. [file Image_1.tiff]

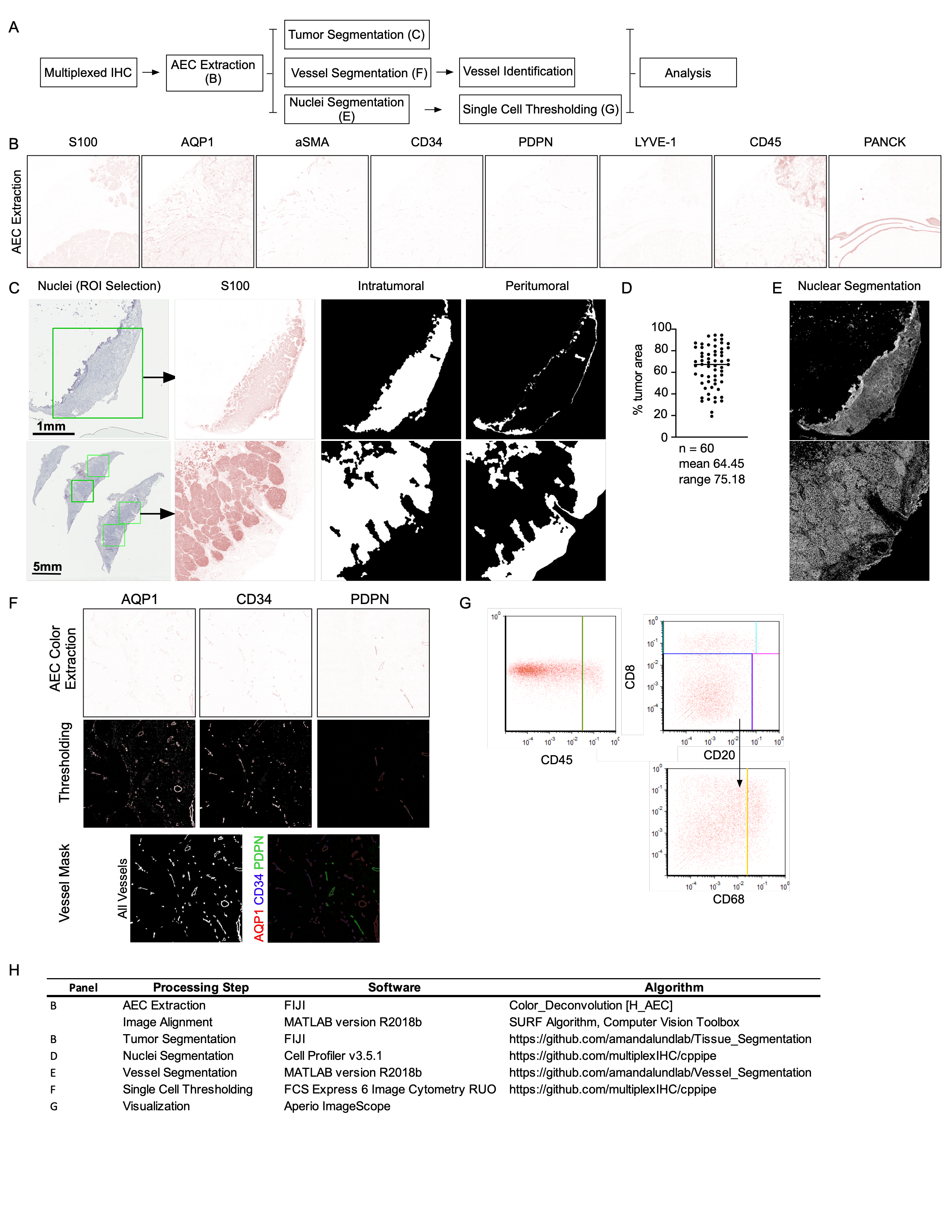

Supplement: Supplementary Figure 2 — Image analysis workflow. (A) Schematic of image analysis workflow. Multiplexed immunohistochemistry (IHC), color (AEC) extraction, image segmentation, single cell and vessel identification and analysis. (B) Representative images of extracted AEC for eight individual markers. (C) Representative images of nuclei scans and ROI selection, S100 staining and peritumoral and intratumoral segmentation masks. (D) Quantitation of percent tumor area per ROI. (E) Representative images of nuclear segmentation from images in (C). (F) Representative images of color extracted (AEC) single stains for AQP1, CD34, and PDPN, binary thresholding, and generation of vessel masks through combination of all three markers. (G) Representative leukocyte gating performed in FCS Express 6 Image Cytometry. (H) Table defining software and packages for each individual analysis step as defined in (A). [file Image_2.tiff]

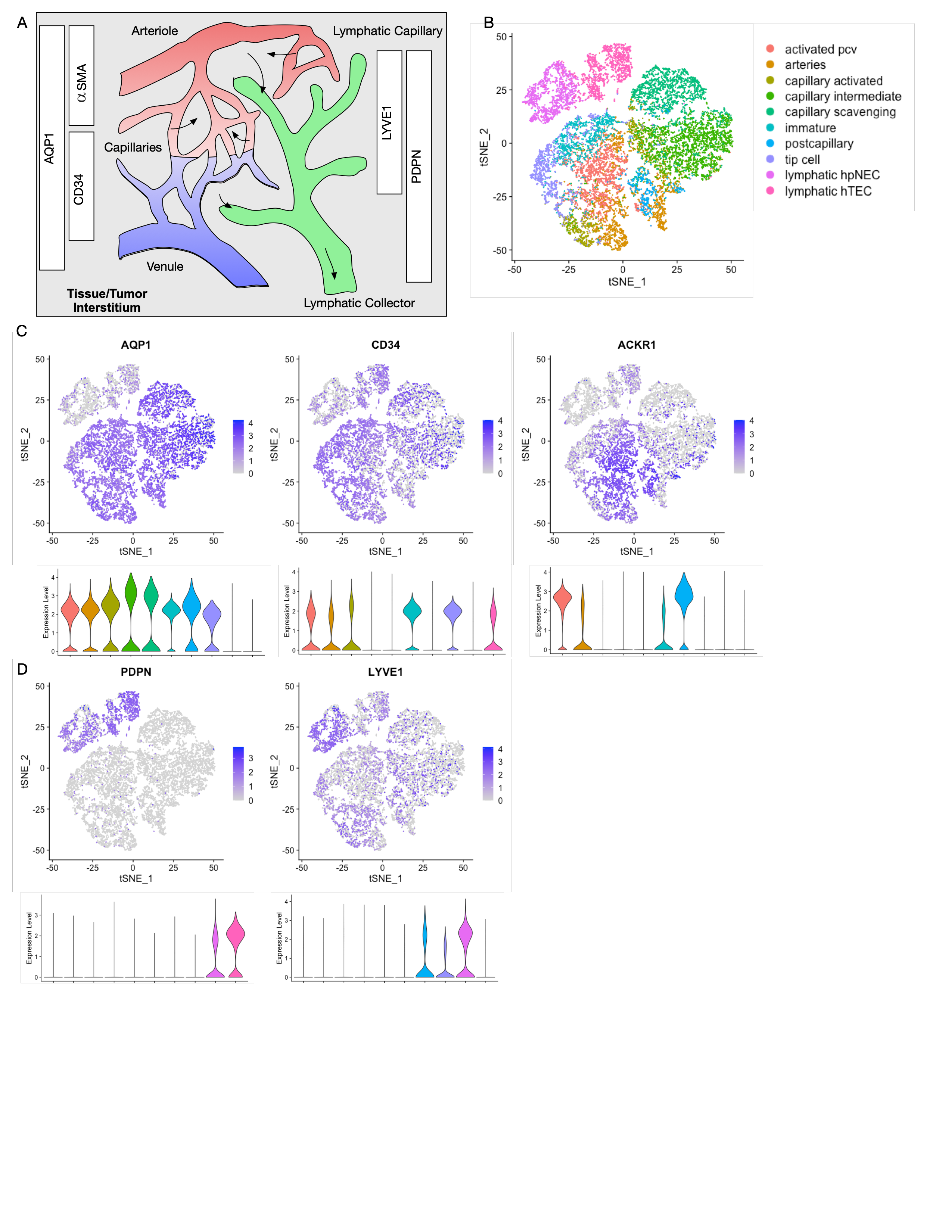

Supplement: Supplementary Figure 3 — Lymphovascular phenotype specification. (A) Schematic representing lymphovascular subtypes identified through imaging workflow. (B) Endothelial subtypes identified by single cell sequencing of CD31+ cells from lung tumors and adjacent normal tissue (analyzed from Goveia et al. Cancer Cell 2020). ACKR1 marker of high endothelial venules. pcv= postcapillary venule; hpNEC=human patient normal endothelial cell; hTEC=human tumor endothelial cell. (C) Blood and (D) lymphatic vessel marker expression across endothelial subtypes. [file Image_3.tiff]

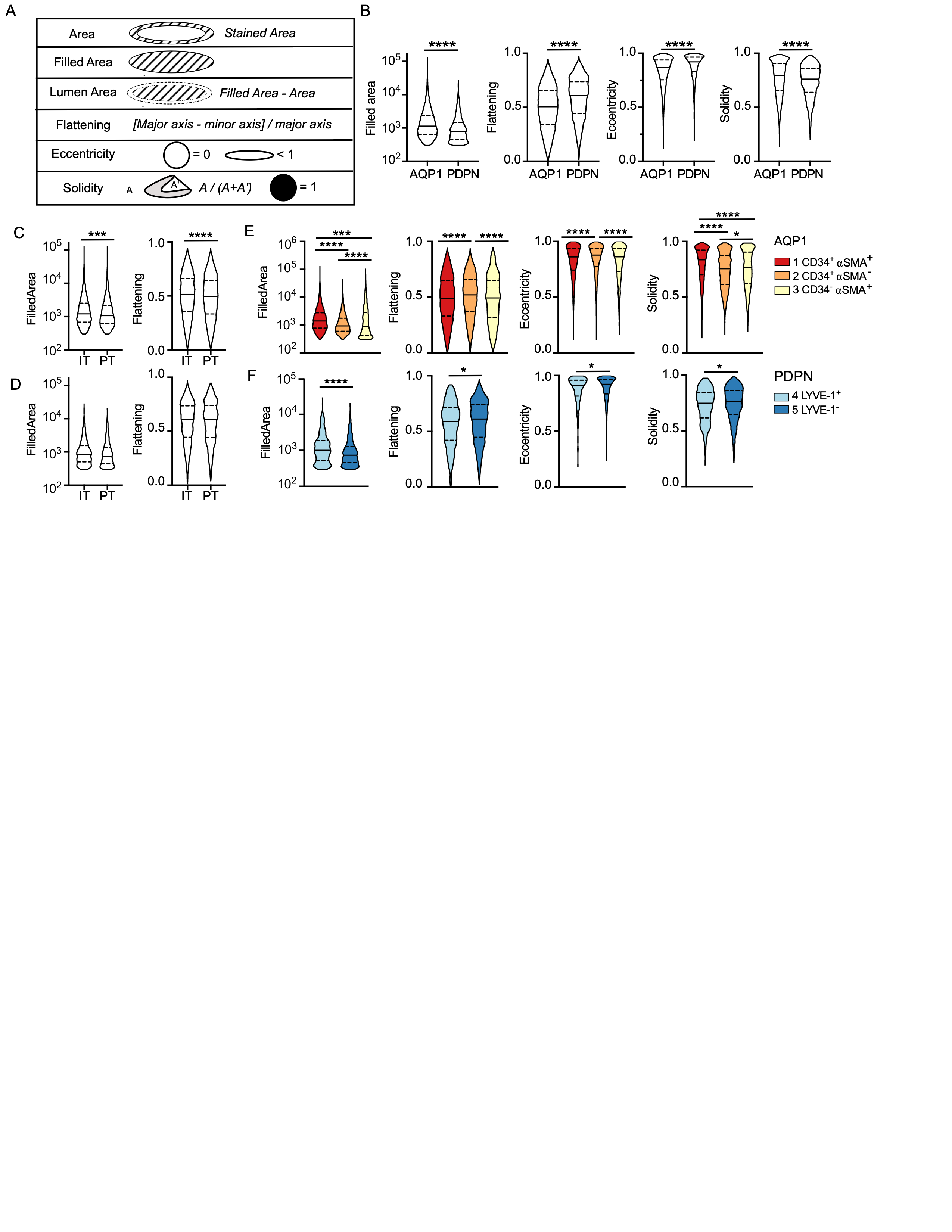

Supplement: Supplementary Figure 4 — Quantitative morphological features of tumor-associated vessels. (A) Schematic explanation of quantitative features extracted from vessel masks. (B) All AQP1 or PDPN positive vessel morphological features across all tumor tissue. (C) AQP1+ vessel morphology in peritumoral (PT) and intratumoral (IT) tumor regions. (D) PDPN+ vessel morphology in PT and IT tumor regions. (E) AQP1+ vessel morphology as a function of subtype. (F) PDPN+ vessel morphology as a function of subtype. Data is representative of individual vessels across samples. Data tested for normality. Mann Whitney test, One-way ANOVA for multiple comparisons. *p<0.05, ***p<0.001, ****p<0.0001. [file Image_4.tiff]

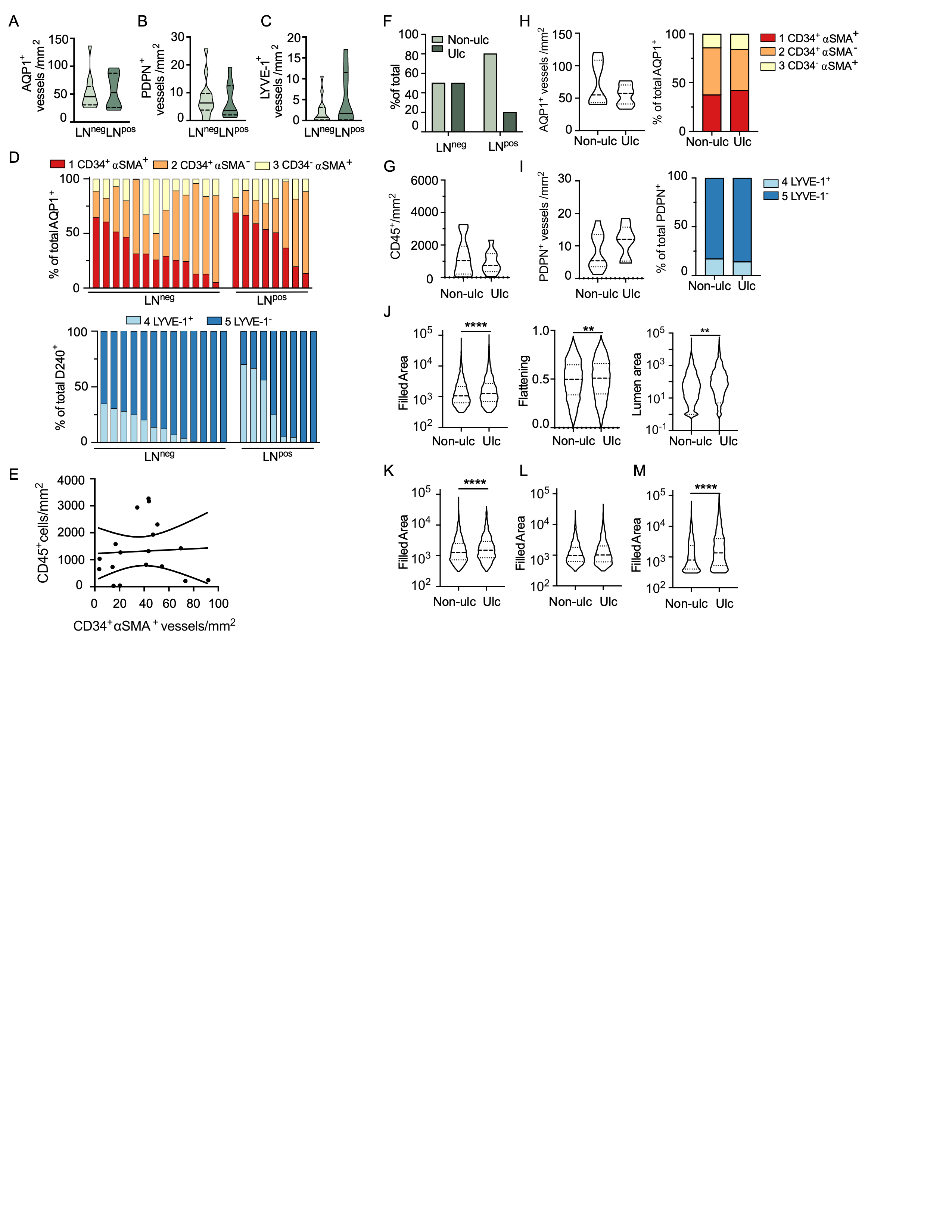

Supplement: Supplementary Figure 5 — Lymphovascular heterogeneity and ulceration in primary melanoma. (A) Intratumoral (IT) AQP1+ blood and (B) PDPN+ lymphatic vessel density. (C) Peritumoral LYVE1+PDPN+ lymphatic vessel density. (D) AQP1+ blood (top) and PDPN+ lymphatic (bottom) vessel heterogeneity per patient in lymph node (LN) positive and negative disease. (E) Correlation between CD45+ cell density and peritumoral AQP1+CD34+αSMA+ immature capillaries. (F) Ulceration (Ulc) as a function of LN status (Non-ulc = non-ulcerated). (G) CD45+ cell density. (H) Total AQP1+ blood vessel density and heterogeneity and (I) PDPN+ lymphatic vessel density and heterogeneity as a function of ulceation. (J) Morphological features (filled area, flattening, and lumen area) of all AQP1+ blood vessels and filled area in (K) CD34+αSMA+, (L) CD34+αSMA-, and (M) CD34-αSMA+ vessel subtypes. Data is representative of individual vessels. Unpaired student’s t-test, **p<0.01, ****p<0.0001. [file Image_5.tiff]

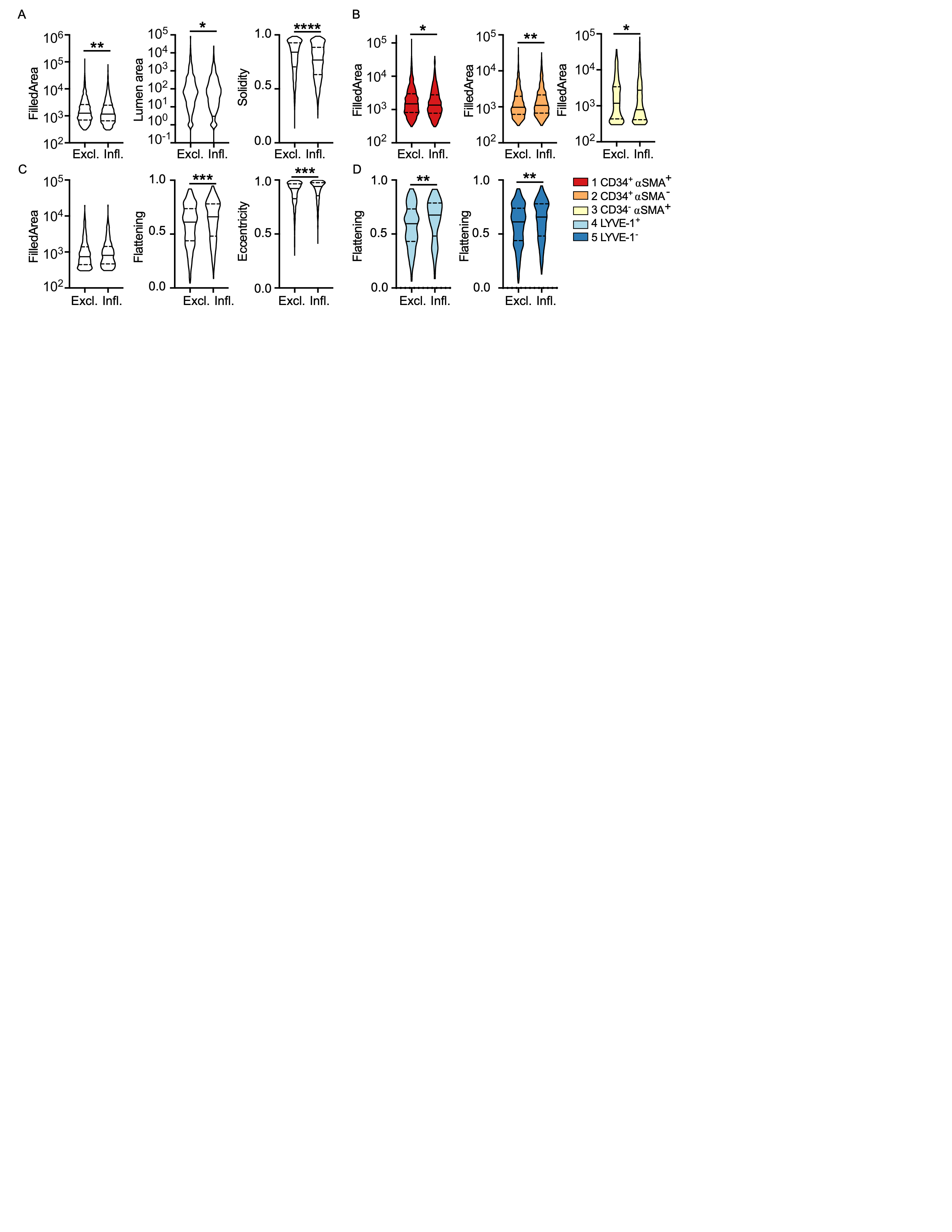

Supplement: Supplementary Figure 6 — Vessel morphology as a function of CD8+ T cell localization in primary melanoma. (A) Intratumoral AQP1+ vessel features in excluded (Excl.) and infiltrated (Infl.) tumors. (B) Vessel size (filled area) as a function of subtype. (C) Peritumoral PDPN+ vessel features in excluded and infiltrated tumors. (D) Vessel flatness as a function of subtype. Data is representative of individual vessels across samples. Data tested for normality. Mann Whitney test, One-way ANOVA for multiple comparisons. *p<0.05, **p<0.01, ***p<0.001, ****p<0.0001. [file Image_6.tiff]
